# Editorial: What Level of Added or Free Sugar Is Commensurate With Good Health Outcomes?

**DOI:** 10.3389/fnut.2021.752534

**Published:** 2021-09-08

**Authors:** Jennie Brand-Miller

**Affiliations:** School of Life and Environmental Sciences and Charles Perkins Centre, The University of Sydney, Sydney, NSW, Australia

**Keywords:** total sugars, added sugars, free sugars, sugar-sweetened beverages, chronic disease, artificial sweeteners

Is there a sweet spot for added sugars? There is consensus that any source of excess calories will contribute to weight gain and metabolic disease, but there is still debate on the level of added or free sugars which is commensurate with both good health and enjoyment of food. While guidelines for the range of energy (%E) as carbohydrate, fat, and protein have widened, the reverse is true of added sugar. In previous decades, health authorities agreed that 10%E was an appropriate upper cut-off, even if strong evidence was lacking. Nonetheless, since 2015, there have been moves to reduce that cut-point to 5%E (1, 2). In the eyes of many, eating as little sugar as possible is ideal.

I have concerns about limiting added (or free sugars) to <5%E. This perspective is informed by knowledge of food science and technology, human evolution, and the role that sweetness plays in encouraging the consumption of healthy foods (e.g., wholegrains). The paradox of falling consumption of added sugars with increasing prevalence of overweight and obesity is now evident in many developed countries (3, 4). In Australia, peak intake of micronutrients is observed within the range 5–15%E from free sugars (5). But of greater concern is the potential of unanticipated and undesirable consequences of health advice on added sugars and sugar-sweetened beverages (SSB). These include the increased incidence of restrictive eating disorders such as orthorexia nervosa (6) and an increase in alcohol consumption and deaths due to alcohol-related disease (7). In Australia, the consumers who avoid SSB, drink twice as many calories in the form of alcoholic beverages as the highest consumers of SSB (8). We should also recall that the history of nutrition science is replete with examples of where we got it wrong, including the “great protein fiasco” (9) and low-fat diets (10).

In this special issue of *Frontiers in Nutrition*, we hoped that the “sweet spot” (the highest level associated with no effect or harm) could be defined with a greater level of certainty. In a well-designed 4-week randomized controlled trial conducted by Te Morenga et al., overweight adults (*n* = 48) randomized to consuming 1,800 kJ of SSB (~100 g added sugar, equivalent to ~1,000 mL of SSB or ~20%E) showed no changes in weight, blood pressure or other cardiometabolic factors compared with those assigned to consuming fruit with a similar energy content (97 g naturally-occurring sugars). However, men (but not women) showed an increase in uricemia, a risk factor for gout. Clearly, further studies in vulnerable groups of similar design and longer duration are needed.

Some studies directly addressed the question of safe levels of intake. In a Swedish population (*n* = 22,877), during a mean follow-up of nearly 20 years, >20%E as added sugar was associated with increased coronary events (HR = 1.39) and stroke risk (HR = 1.31) compared to 7.5–10%E as added sugar (Janzi et al.). Surprisingly, participants with the *lowest* intake (<5%E) had the *highest* risk of atrial fibrillation and aortic stenosis. This result is difficult to explain but emphasizes the complexity of nutrition and observational studies. In a well-characterized older cohort of Australians (*n* = 1,713), changes in the %E from added sugars were not associated over time with changes in body weight, regardless of source—beverage or non-beverage (Moshtaghian et al.). Earlier work in this cohort found those consuming <5%E as added sugar, were higher consumers of alcohol (11).

Added sugars are used in tiny amounts (~1–2% w/w) to enhance flavors and improve palatability. If this encourages excessive energy intake, then the same must be said of salt, herbs, spices, and soy sauce, all of them in use for thousands of years. In large amounts, the physicochemical, technological, and functional characteristics of sugars influence human metabolism as reviewed by Brouns. Sugars also influence dental health. The effect of sugars on oral health was the conditional reason that WHO recommended consumption of free sugars to below 5% of total energy (2). But Brouns reminds us that frequency, contact time, and rapidly digestible starches and acidic foods like wine and fruit also affect dental health. Reducing the amount of added sugars from SSB will not be effective if starchy snacks and sticky confectionery are consumed instead (3).

Determining the extent to which added sugars contribute to disease in various populations is challenging because it is difficult to accurately measure intakes. Biomarkers of sugar intake may therefore be helpful although this does not distinguish added sugars from sugars in fruit and vegetables. Te Morenga's second paper (Te Morenga et al.) found that the sum of urinary [sucrose + fructose] was weakly but significantly correlated (r = 0.23) with intakes of total sugars and with added sugars from SSB (*n* = 0.26). Interestingly, they found a higher correlation (r = 0.40) with the C-13 carbon isotope ratio of alanine. Similarly, in the DONALD study (*n* = 254 adolescents), Della Della Corte et al. backed up dietary records with measurement of fructose and the sum of [fructose + sucrose] in two complete 24-h urine collections. They found no prospective associations between adolescent intake of fructose, sucrose, glucose, added, free, and total sugar with adult insulin sensitivity as measured by HOMA2-%S. Indeed, higher fructose in urine was associated with *improved* insulin sensitivity in females (but not males).

An underlying assumption of recommendations to reduce SSBs, is that water will take their place. In the US population (NHANES, *n* = 22,716), Drewnoski's group reported that SSB consumption had declined by ~20% in volume between 2011 and 2016 (Vieux et al.), whereas plain and bottled water increased by just ~10%. The opposing time trends were not uniform—lower income and minority groups consumed more bottled water and relatively little tap water. In this context, changes in intake of alcoholic beverages and other sources of energy (e.g., chocolate) must be explored.

Pang et al. attracted the highest number of views with their review of the current state of knowledge on artificial sweeteners, reminding us that they are not all the same, with different chemical structures, absorption, and metabolic effects. Despite many being in use for 50 years, there are still very few long-term studies to show that substituting sugars and SSB with non-caloric alternatives is of benefit. And finally, an updated meta-analysis and systematic review by Zafar et al. confirmed that chronic consumption of fructose is neither more beneficial nor harmful than sucrose or glucose for glycemia and other metabolic outcomes.

Taken together, this collection of 11 papers provides evidence that a diet containing >20% added sugars may have adverse effects, but so too, a diet containing <5% added sugar. At worst, such a restrictive diet can create food fear or an unhealthy relationship with food and alcohol, especially for women and girls. As the Swedish study found (Janzi et al.), the sweet spot may therefore lie somewhere between 7.5 and 10%E as added sugars. Many will agree that public health interventions and food taxes to prevent obesity and related diseases should promote the quality of the overall diet, not a singular focus on reducing sugar and SSB intakes.

## Author Contributions

The author confirms being the sole contributor of this work and has approved it for publication.

## Funding

JB-M is a recipient of past and current National Health and Medical Research funding from the government of Australia.

## Conflict of Interest

JB-M is President of the Glycemic Index Foundation and overseas a glycemic index testing service at the University of Sydney. She receives royalties from the University of Sydney and popular books about nutrition and health.

## Publisher's Note

All claims expressed in this article are solely those of the authors and do not necessarily represent those of their affiliated organizations, or those of the publisher, the editors and the reviewers. Any product that may be evaluated in this article, or claim that may be made by its manufacturer, is not guaranteed or endorsed by the publisher.
